# Healthy soils for healthy plants for healthy humans: How beneficial microbes in the soil, food and gut are interconnected and how agriculture can contribute to human health

**DOI:** 10.15252/embr.202051069

**Published:** 2020-07-31

**Authors:** Heribert Hirt

**Affiliations:** ^1^ DARWIN21 Center for Desert Agriculture King Abdullah University of Science and Technology Thuwal Saudi Arabia; ^2^ Max F. Perutz Laboratories University of Vienna Vienna Austria

**Keywords:** Ecology, Microbiology, Virology & Host Pathogen Interaction, Plant Biology

## Abstract

The microbiota of the human gut and the plant rhizome are similar in many ways and intricately connected with each other. A healthy plant therefore affects human microbiota and human health.

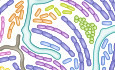

The human gut microbiome is a complex system of gazillions of bacteria, fungi, viruses, protists and archaea that has an enormous effect on our metabolism, health and well‐being. The same holds true for the plant rhizosphere, the crucial parts below ground: roots are immersed in a soil microbiome that provides plants with important nutrients, protects them from disease and pathogens and helps plants to adapt to environmental changes (Fig 1). And, similar to faecal transplants in humans, soil transplants can have a drastic effect on plant health and growth. Moreover, plant and human microbiomes are linked to each other: since the gut and the soil microbiome share similar bacteria phyla and since microbes from fruits, salads and vegetables join the human gut microbiome, the plant microbiome can affect the gut microbiome and thereby human health (Fig 2). The current and well‐known concept of a healthy died—one that includes a lot of fibre, minerals and vitamins from fruits and vegetables—should therefore be expanded to consider plant microbes that not only benefit plant health but via food also human health. Vice versa, as much as antibiotics can severely change the human gut microbiome and its function, the use of herbicides, fungicides and pesticides in food production has drastic effects on the plant microbiomes in the soil and on the fruits and vegetables that we eat.

**Figure 1:**
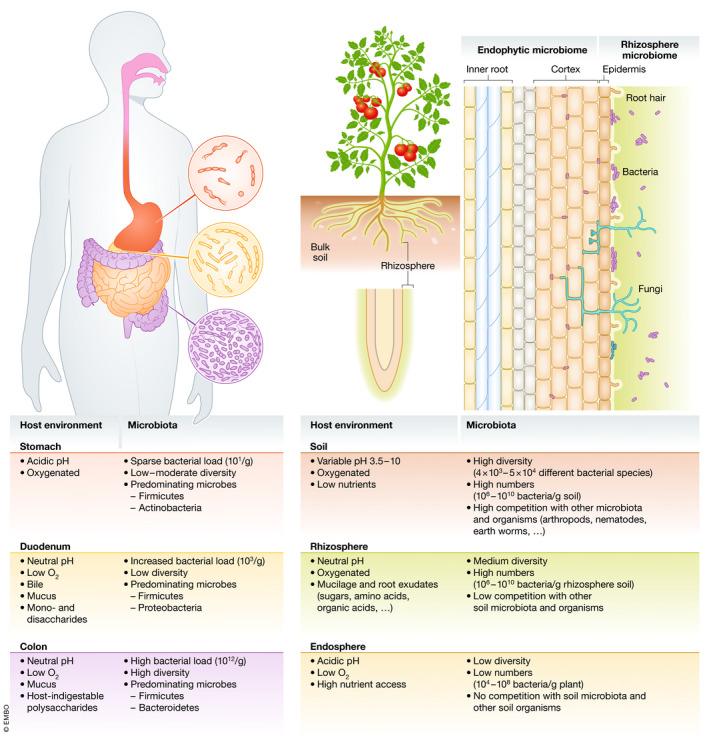
**The gut microbiota in humans and the soil and rhizome microbiota in plants exist under similar environmental conditions.**

**Figure 2:**
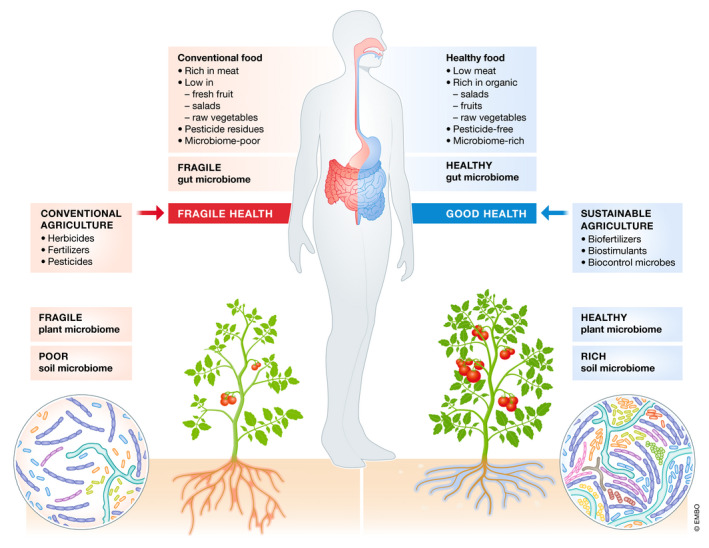
**The direct and indirect effects of the plant microbiota on the human gut microbiome.**

## The gut microbiome

A human body consists of about ten times more bacterial cells than human cells, the majority of which are in the gut. The ratio of microbial to human genes is even more impressive, counting more than 3 million microbial genes compared with 22,000 human genes. The gut microbiome starts to develop before birth and becomes fully established 2–3 years into childhood. The formation of the infant microbiome is not only important for gut function, but also crucial for the development of the systemic and mucosal immune system thereby influencing infant and eventually adult health (Lozupone *et al*, 2012).

… since microbes from fruits, salads and vegetables join the human gut microbiome, the plant microbiome can affect the gut microbiome and thereby human health.

The original view of a simple mutualistic interaction between gut cells and microbial cells has given way to a much more complex and dynamic view of a close symbiotic interaction between humans and bacteria. The intestinal epithelial and mucosal immune cells recognize and interact with select bacterial species which contribute to the proper functioning of the human immune system. Microbially generated metabolites not only help the gut to extract nutrients from food, but can also influence immune function (Postler & Ghosh, 2017). In fact, a dysfunctional gut microbiome has been shown to cause or contribute to various gastrointestinal diseases, inflammatory or immune‐mediated diseases, diabetes, obesity, atopic diseases and chronic kidney diseases (Lozupone *et al*, 2012). Generally, microbiome richness and diversity are directly associated with human health, but this simple equation needs to be considered with care.

An important step towards our current understanding was the finding that healthy and sick gut microbiomes differ in their microbial composition. Although gut microbiomes contain up to 1,000 different microbial species and show large variations between individuals, 99% of the gut microbiota belongs to only 30–40 species (Lozupone *et al*, 2012) that change in positive or negative ways in response to external or environmental factors. Novel sequencing techniques now allow the detection and quantification of virtually all gut microbes, but we still know almost nothing about the role and function of many microbial species, let alone the role of viruses that also populate the gut ecosystem.

## Changes of the microbiota in historical times

As humans and human civilization changed over millennia so did the human gut microbiota in response to changes in diet. The gut microbiome of contemporary hunter–gatherer societies for instance shows drastic changes during the year reflecting the changes in food supply. Moreover, major differences can also be observed between the microbiota of female and male members of these societies: the microbiota of women resembles more those of herbivores, while the male members have a more carnivore‐like microbiome. The changes in gut microbiota from earlier to modern civilizations also reflect changes in hygiene, which can still be observed between urban and rural communities. Modern lifestyle with improved hygiene, processed food and the widespread use of medicines, notably antibiotics, seems to have had a major effect on human gut microbiome diversity during the past decades, overall reducing its variety.

Importantly, what people eat has a much stronger influence on the gut microbial composition than genetics: members of the same family living in different locations show larger differences in their microbiomes than genetically unrelated individuals who share the same household and similar lifestyle and nutrition.

## Microbes enhance food quality and content

Humans can only synthesize 11 of the 20 essential amino acids themselves; they rely on food intake for the other nine along with all 13 essential vitamins. Most of these amino acids and vitamins are retrieved from meat, eggs, milk products, fruits and vegetables, but a few essential compounds are produced by microbes—which are important producers of essential amino acids and vitamins themselves. For example, cobalamin (Vitamin B12) cannot be produced by either plants or animals; it is synthesized by microbes in the plant microbiotas or in the gut of ruminant animals.

In addition to primary metabolites, amino acids and vitamins, many microbes also produce a large variety of chemicals known as secondary metabolites or natural products. Among the best‐known of these compounds are antibiotics but also immunosuppressants, anticancer and anti‐inflammatory drugs.

Yet, plants are at least as capable as microbes in producing secondary metabolites; overall plants synthesize more than hundred thousand compounds, many of which are used as pharmaceuticals or are important for human health. Flavonoids, a highly diverse class of plant compounds that are present in many fruits, vegetables or nuts, have many biological activities including anti‐inflammatory, anticancer and anti‐viral properties. Omega‐3 (n‐3) polyunsaturated fatty acids (PUFA) are found in nuts and seeds of twenty different plants, including soy bean, rape seed or flax. PUFA reduce the risk of cardiovascular diseases, blood pressure and inflammatory reactions. Another class of important plant products are conjugated linoleic acid, L‐carnitine, choline or sphingomyelin, which all positively affect the gut microbiome (Postler & Ghosh, 2017). Interestingly, many plants produce only tiny amounts of these secondary metabolites, but beneficial microbes associated with their plant host can boost their production. The interaction of microbes and plants thereby influences food quality, taste and texture (Flandroy *et al*, 2018).

## Where does our food come from?

Food production has changed tremendously during the past century. Today's agricultural production systems are mostly large‐scale monocultures of a few elite crop varieties that require fertilizers, herbicides and pesticides to ensure a high yield. Most of these high‐yield breeds have lost important secondary metabolites that protect plants and humans alike. A good example is the domestication of plants of the *Brassicacae* family, such as cabbage and cauliflower, in which the amount of glucosinolates has been reduced to eliminate their bitter taste. Yet, glucosinolates not only help the plant to resist to pathogens but are also suspected to be a prebiotic anticancer metabolite (Blum *et al*, 2019).

Modern lifestyle with improved hygiene, processed food and the widespread use of medicines, […] seems to have had a major effect on human gut microbiome diversity during the past decades

Industrial agriculture requires increasing amounts of fertilizers and pesticides to maintain yield. This seems to be the result and/or the cause of a poor microbial diversity in the soil. Soil erosion and climate change also affect microbial biodiversity and contribute to the loss of large areas of arable land and their microbial populations (Blum *et al*, 2019). In this way, crop plants today lack many of their important symbiotic partners to produce or increase the contents of vitamins, minerals, antioxidants and other metabolites that are beneficial for both plant and human health.

Soil is the ultimate source from which plants recruit beneficial microbes for the rhizosphere and phyllosphere, that is the root and shoot surfaces, but also for the inner plant organs (endosphere), including fruits and seeds. Plant rhizo‐, phyllo‐ and endosphere microbes not only increase nutrient use efficiency and thereby crop yield, they are also involved in enhancing resistance against herbivores, insects, bacterial and fungal pathogens and even nematodes or viral infections (Blum *et al*, 2019).

The use of herbicides, excessive mineral fertilization and improper land management have serious effects on microbial communities. A good example is glyphosate that has been used for more than 40 years in agriculture. This chemical inhibits enoylpyruvylshikimate‐5‐phosphate (EPSP) synthase, an enzyme of the shikimate pathway that is responsible for the biosynthesis of aromatic amino acids in plants. EPSP synthase is present in all plants but not in humans, which makes glyphosate an ideal herbicide. The application of glyphosate to kill weeds is linked with the use of glyphosate‐resistant crops which has helped considerably to assure high crop yields.

But the use of glyphosate might come with a price. Although the acute toxicity of glyphosate to humans is low, the fact that humans are exposed to it over long terms prompted the WHO to classify glyphosate as a potential carcinogenic in 2015. Importantly, glyphosate is also an antimicrobial, as both bacteria and fungi rely on the shikimate pathway for aromatic amino acid production. A number of reports show negative effects on beneficial soil, rhizosphere and endosphere microbes, including arbuscular mycorrhizal fungi and nitrogen‐fixing *Rhizobium spp*. (Van Bruggen *et al*, 2018). Glyphosate also seems to inhibit a number of soil, plant and gut beneficial microbes at much lower concentrations than pathogenic microbes. In terms of the human gut microbiome, such inhibition was observed for the beneficial microbes *Bifidobacterium* sp. and *Enterococcus* sp. compared with pathogenic strains of *Clostridium* sp. and *Salmonella* sp. (Van Bruggen *et al*, 2018). Overall these indirect effects of glyphosate on soil, plant and human microbes might affect human health.

## Food quality beyond fibres, minerals and vitamins

The protein‐rich input from increased meat consumption in Western diets also massively affects the gut microbiome, whereby certain microbes suppress beneficial competitors and change our eating behaviour to favour more unhealthy food. Much of the current discussion on maintaining a diverse and healthy gut microbiome is focused on eating a healthy diet, which is defined by a high content of fibre, minerals and vitamins. However, this still leaves out an important aspect of food.

Most of our daily food comes from industrial agriculture and has been exposed to herbicides, fertilizers and a large array of pesticides to obtain high yields. Pesticides are a large class of chemical compounds that include fungicides, bactericides, nematicides, molluscicides, avicides, rodenticides and animal repellents. A large literature is available to show the negative effects of many commonly used pesticides on human health. For example, various carbamates, pyrethroids and neonicotinides have endocrine‐disrupting activity and negative effects on reproduction in animals and humans (Nicolopoulou‐Stamati *et al*, 2016). However, many beneficial microbes are also among the targets of pesticides with direct and indirect implications on soil, plant and food safety.

The interaction of microbes and plants thereby influences food quality, taste and texture.

For example, most copper‐based fungicides have a deleterious effect on nitrogen‐fixing bacteria (Meena *et al*, 2020). Similarly, long‐term application of organomercurials has negative effects on cellulolytic fungal species. Triarimol and captan decrease the content of *Aspergillus fungi* that help plants to grow and develop. Carbendazim is highly toxic to *Trichoderma harzianum*, a potent biocontrol agent against the soil‐borne fungal pathogens *Fusarium*,* Pythium* and *Rhizoctonia* and many fungicides also inhibit hyphal growth and root colonization by arbuscular mycorrhizal fungi. The insecticides chlorpyrifos, phosphamidon, malathion, fenthion, methyl phosphorothioate, parathion, chlorfluazuron, cypermethrin or phoximin have negative effects on soil and rhizosphere microbiota at field‐recommended concentrations (Meena *et al*, 2020).

Many fresh fruit, salads and vegetables are stored and shipped, often over long distances, before they arrive at the supermarket. Long storage and shipping periods, however, are not possible without treating fruit and vegetables with a variety of pesticides and antibiotics for preservation. Not only will some of these chemicals make their way through food into the human gut, but they also kill off the plant microbiota.

Agriculture uses about four times more antibiotics than human medicine. This massive (ab)use of antibiotics in farming, mostly to enhance growth and health of livestock, has greatly contributed to the emergence of resistant bacteria. Not only do antibiotics excreted by animals change microbial function and composition of soil, waterways and other biotopes but also the antibiotic resistance genes can spread to other microbes via horizontal gene transfer (Jechalke *et al*, 2014). The consumption of fresh produce from fields fertilized with manure from antibiotics‐treated animals can thus spread resistance genes to the human gut microbiome and further the emergence of multi‐drug‐resistant human pathogens. The widespread application of pesticides and herbicides could similarly increase the risk of new pathogens and diseases against both plants and humans.

… crop plants today lack many of their important symbiotic partners to produce or increase the contents of vitamins, minerals, antioxidants and other metabolites…

## Similarities between root and gut microbiomes

Recent research suggests that the root and gut microbial communities exist under similar conditions (Mendes & Raaijmakers, 2015). Both are open systems characterized by gradients of oxygen, water and pH that create a diversity of different niches. Both systems inherit their microbial members from the environment: food in humans and soil in plants, respectively. Plant and gut systems are populated by a multitude of similar bacterial phyla (Firmicutes, Bacteroidetes, Proteobacteria, Actinobacteria) and, similar to human faecal transfer, transplantation of beneficial microbes from disease‐suppressive soils can protect plants against various diseases (Mendes & Raaijmakers, 2015). Research on different mammalian herbivores and carnivores indicates that the gut microbiome recruits some of its members from eating raw plant material. Root and gut microbes synthesize essential amino acids, vitamins and many other secondary metabolites that modulate their host immune system: as such, the plant and gut microbiomes can be considered as meta‐organs with paramount importance for the health of their hosts.

Most of our daily food comes from industrial agriculture and has been exposed to herbicides, fertilizers and a large array of pesticides to obtain high yields.

It is therefore important to better understand the functions and roles of the hundreds of different microbial species in the complex interaction network with their hosts. Of equal importance is the question how to establish and maintain a healthy microbiome. At the same time, the re‐integration of beneficial microbes into agriculture could contribute to providing healthy food in a sustainable manner so as to help reduce the amount of fertilizer, pesticides and herbicides being used (Bender *et al*, 2016). Moreover, given the food link, humans would also benefit from eating unprocessed organic food since it supplies beneficial microbes along with secondary metabolites. Research on the integral role of microbiomes on their host's metabolism and health should therefore not stop at the human gut microbiome but expand to the microbiota of plants and their function in plant growth and development. Given the food link, such an effort would benefit both plants and humans.
